# Genome Sequence of Bacillus vallismortis TD3, a Salt-Tolerant Strain Isolated from the Sediments of a Solar Saltern in Tamil Nadu, India

**DOI:** 10.1128/MRA.00817-18

**Published:** 2018-07-12

**Authors:** Chandrasekaran Suganthi, Anbazhagan Mageswari, Manoharan Shankar, Kodiveri M. Gothandam, Sivashanmugam Karthikeyan

**Affiliations:** a School of Bio-Sciences and Technology, Vellore Institute of Technology, Vellore, Tamil Nadu, India; Queens College

## Abstract

Various Bacillus spp. capable of producing enzymes with industrially desirable properties have been isolated from adverse environments. Here, we announce the 3.91-Mbp draft genome sequence of a moderately salt-resistant Bacillus vallismortis strain, TD3, capable of producing several industrially relevant enzymes.

## Abstract (precis)

Various Bacillus spp. capable of producing enzymes with industrially desirable properties have been isolated from adverse environments.

## ANNOUNCEMENT

Bacteria colonizing under adverse environmental conditions are potential sources for bioprospecting industrially important enzymes with specific characteristics (1). Bacillus is one of the most popular bacterial genera that is frequently detected in various environments, including hostile habitats. For example, extremophilic Bacillus spp. that have adapted to live in environments such as hydrothermal vents (2), the permafrost (3), heavy-metal-contaminated areas (4), oil-contaminated waters (5), and high-salinity environments (6) have been reported earlier. From an industrial perspective, halophilic bacteria are attractive candidates for exploitation, as their enzymes have also adapted to function under adverse reaction conditions (7). Our lab has also identified and characterized industrially important enzymes from halotolerant bacteria (8, 9).

Isolate TD3 was originally recovered from the sediments of a solar saltern at Tuticorin, Tamil Nadu, India, which is a port town located in the Coromandel Coast of the Bay of Bengal. TD3 was a Gram-positive motile rod, which was moderately halophilic, with the ability to grow easily in the presence of 10% (wt/vol) NaCl. Isolate TD3 was identified as Bacillus vallismortis by partial sequencing and analysis of its 16S rRNA gene. Total genomic DNA from B. vallismortis TD3 was isolated using the HiPurA bacterial genomic DNA purification kit (HiMedia, Mumbai, India), and the genome sequence of B. vallismortis TD3 was generated at Genotypic Technology, Bangalore, India, by Illumina sequencing. Illumina paired-end libraries were constructed per manufacturer-recommended protocols, targeting a read length of 100 bp, and were sequenced on a HiSeq system. The resulting reads were subjected to quality control using SeqQC version 2.2 (Genotypic Technology, Bangalore, India) for adapter trimming, B trimming, and low-quality end trimming. The remaining high-quality reads were assembled *de novo* using SPAdes version 3.1.0 (10), generating 152 contigs yielding a total length of 3,914,588 bp and an *N*_50_ value of 228,120 bp. These 152 contigs were then subjected to scaffolding using SSPACE version 2.0 (11), yielding a final sequence length of 3,912,114 bp in a set of 29 scaffolds, with a final *N*_50_ value of 258,393 bp.

Coding sequences in the B. vallismortis TD3 genome, which had a GC content of 43.9%, were predicted using the Rapid Annotations using Subsystems Technology (RAST) server (12). A total of 4,206 genes were predicted, including those coding for 113 RNAs (rRNA and tRNA). B. vallismortis TD3 encoded osmotolerance determinants mostly restricted to the accumulation of compatible solutes, as opposed to the accumulation of salts in the cytoplasm, as is typical of Halobacterium species. We detected genes encoding mechanisms for the uptake of K^+^ ions, glycine-betaine, carnitine, and proline, all of which are known to be involved in the response to osmotic stress (13). We also identified genes coding for α-amylase, pectin lyase, endoglucanase, several proteases, and a β-glucosidase. We are currently exploring the characteristics of these enzymes and their potential applications in relevant industries. Additionally, we are also pursuing the genes and proteins that contribute to the various molecular mechanisms of halotolerance in B. vallismortis TD3.

### Data availability.

This whole-genome shotgun project has been deposited in DDBJ/ENA/GenBank under the accession no. NXEM00000000. The version described in this paper is the first version, NXEM01000000.
